# Liver function test abnormalities in a longitudinal cohort of Thai individuals treated since acute HIV infection

**DOI:** 10.1002/jia2.25444

**Published:** 2020-01-17

**Authors:** Michael J Peluso, Donn J Colby, Suteeraporn Pinyakorn, Sasiwimol Ubolyam, Jintana Intasan, Rapee Trichavaroj, Nitiya Chomchey, Peeriya Prueksakaew, Bonnie M Slike, Shelly J Krebs, Ningbo Jian, Merlin L Robb, Praphan Phanuphak, Nittaya Phanuphak, Serena Spudich, Jintanat Ananworanich, Eugène Kroon, on behalf of the SEARCH010/RV254 Study Group, Nipat Teeratakulpisarn, Supanit Pattanachaiwit, Carlo Sacdalan, Somchai Sriplienchan, Mark de Souza, Ponpen Tantivitayakul, Kultida Poltavee, Tassanee Luekasemsuk, Hathairat Savadsuk, Somporn Tipsuk, Suwanna Puttamsawin, Khunthalee Benjapornpong, Nisakorn Ratnaratorn, Kamonkan Tangnaree, Chutharat Munkong, Rommanus Thaimanee, Patcharin Eamyoung, Supranee Buranapraditkun, Sukalya Lerdlum, Sopark Manasnayakorn, Rugsun Rerknimitr, Sunee Sirivichayakul, Phandee Wattanaboonyongcharoen, Duanghathai Suttichom, Robert O'Connell, Alexandra Schuetz, Denise Hsu, Siriwat Akapirat, Bessara Nuntapinit, Nantana Tantibul, Nampueng Churikanont, Saowanit Getchalarat, Nelson Michael, Sandhya Vasan, Trevor Crowell, Ellen Turk, Corinne McCullough, Oratai Butterworth, Mark Milazzo, Leigh Anne Eller

**Affiliations:** ^1^ Division of HIV, Infectious Diseases, and Global Medicine University of California San Francisco CA USA; ^2^ SEARCH Thai Red Cross AIDS Research Centre Bangkok Thailand; ^3^ U.S. Military HIV Research Program Walter Reed Army Institute of Research Silver Spring MD USA; ^4^ Henry M. Jackson Foundation for the Advancement of Military Medicine Bethesda MD USA; ^5^ HIV‐NAT Thai Red Cross AIDS Research Centre Bangkok Thailand; ^6^ Armed Forces Research Institute of Medical Sciences Bangkok Thailand; ^7^ Yale University School of Medicine New Haven CT USA; ^8^ The University of Amsterdam Amsterdam The Netherlands

**Keywords:** HIV, acute HIV, liver function tests, Acquired Immunodeficiency Syndrome, antiretroviral agents, anti‐HIV agents, Thailand

## Abstract

**Introduction:**

Liver disease is a common cause of non‐AIDS morbidity and mortality in people living with HIV (PLHIV), but the prevalence and significance of liver function test (LFT) abnormalities in early HIV infection is unknown. This study aimed to characterize LFTs in a large cohort of participants with acute HIV infection initiating immediate antiretroviral therapy (ART) and examine the association between LFTs and biomarkers of HIV infection and inflammation.

**Methods:**

We measured LFTs at the time of HIV diagnosis and at 4, 12, 24 and 48 weeks after ART initiation in 426 Thai individuals with acute HIV infection from 2009 to 2018. A subset of individuals had data available at 96 and 144 weeks. We excluded individuals with concomitant viral hepatitis. Alanine aminotransferase (ALT) was the primary outcome of interest; values greater than 1.25 times the upper limit of normal were considered elevated. Analyses utilized descriptive statistics, non‐parametric tests and multivariate logistic regression.

**Results:**

Sixty‐six of the 426 individuals (15.5%) had abnormal baseline ALT levels; the majority (43/66, 65.5%) had Grade 1 elevations. Elevated baseline ALT correlated with Fiebig stages III to V (*p* = 0.001) and baseline HIV RNA >6 log_10_ copies/mL (*p* = 0.012). Baseline elevations resolved by 48 weeks on ART in 59 of the 66 individuals (89%). ALT elevations at 24 and 48 weeks correlated with Fiebig stages I to II at diagnosis (*p* < 0.001), baseline plasma HIV RNA levels <6 log_10_ copies/mL (*p* < 0.001), abnormal baseline ALT (*p* < 0.001), baseline CD4 >350 cells/μL (*p* = 0.03) and older age (*p* = 0.03). Individuals initiating efavirenz‐based regimens were more likely to have elevated ALT levels at 48 weeks compared with those on non‐efavirenz‐based regimens (*p* = 0.003).

**Conclusions:**

One in six people with acute HIV infection have elevated LFTs. Clinical outcomes with ART started in acute HIV are generally good, with resolution of ALT elevations within 48 weeks on ART in most cases. These results suggest a multifactorial model for hepatic injury involving a combination of HIV‐associated and ART‐associated processes, which may change over time.

## 1 Introduction

Liver disease is a common cause of non‐AIDS related morbidity and mortality in people living with HIV (PLHIV) 1. In the era of modern antiretroviral therapy (ART), the spectrum of liver disease in PLHIV has shifted from opportunistic infections to the sequelae of chronic infection, cumulative medication toxicity 2, and comorbidities including viral hepatitis, alcohol toxicity and fatty liver disease 3, 4, 5. While abnormalities in liver function tests (LFTs) have been identified as a feature of primary HIV infection in case reports and smaller cross‐sectional cohorts 6, 7, the incidence, time course and long‐term consequences of LFT perturbations following ART initiation during early infection have not been described in detail.

In this study, we longitudinally characterize LFTs in a large cohort of participants with acute HIV infection (AHI) who initiated immediate ART and examine the association between LFTs and biomarkers of HIV infection and inflammation.

## 2 Methods

This analysis took place within the SEARCH010/RV254 cohort (https://clinicaltrials.gov NCT00796146) and included Thai participants diagnosed with AHI between 2009 and 2018. Screening for AHI was performed using pooled nucleic acid testing (NAT) and sequential HIV enzyme immunoassay (EIA) in accordance with previously published methods 8, 9. AHI was defined by either a non‐reactive fourth‐generation EIA with a positive nucleic acid test or reactive fourth‐generation EIA with a non‐reactive second‐generation EIA. Individuals with viral hepatitis co‐infection (hepatitis A, B or C; n = 45) identified at screening or follow‐up were excluded from the analysis.

The stage of HIV infection was determined using the fourth‐generation (4^th^G) acute infection staging 9 and Fiebig systems 10. Enrolled participants completed a clinical interview, physical examination and blood draw including LFTs at baseline. These were each repeated at four, twelve, twenty‐four and fourty‐eight weeks after study entry. In all, 426 ART‐naïve Thai adults with AHI were included in the primary analysis up to the 48‐week endpoint. A subset of individuals had data available at 96 and 144 weeks (n = 278 and n = 282 respectively).

Participants initiated ART within 24 to 72 hours of the baseline assessment. From 2009 to 2016 the standard first‐line ART regimen was efavirenz plus two nucleoside reverse transcriptase inhibitors (NRTIs). Subsets of participants were randomized to receive “mega‐ART,” composed of standard ART with the addition of maraviroc or maraviroc plus raltegravir. In February 2017, the standard first‐line ART regimen was changed to dolutegravir plus two NRTIs. Substitutions could be made in individual drugs for clinical indications such as intolerance or resistance. Because of the association between non‐nucleoside reverse transcriptase inhibitor (NNRTI)‐based regimens and drug‐induced liver injury 2, 11, the primary ART‐related outcome of interest was LFT differences in individuals receiving efavirenz‐containing (n = 373) or efavirenz‐sparing (n = 53) regimens as their initial ART regimen.

Plasma HIV RNA was measured using either the Roche Amplicor HIV‐1 Monitor Test v1.5 or the Roche COBAS AmpliPrep/COBAS TaqMan HIV‐1 Test v2.0 (Roche Diagnostics, Branchburg, New Jersey, USA). Lower limits of detection were 50 and 20 copies/mL respectively; analyses utilized 50 copies/mL as the lower limit of detection. Standard laboratory tests included CD4^+^ and CD8^+^ T lymphocyte count, complete blood count (CBC), alanine aminotransferase (ALT), gamma‐glutamyl transferase (GGT), total and direct bilirubin. Values of ALT, GGT and bilirubin were compared with the reference value for the upper limit of normal (ULN) for the assay (ULN for males: ALT 55 U/L, GGT 54 U/L, total bilirubin 1.2 mg/dL; ULN for females: ALT 55 U/L, GGT 36 U/L, total bilirubin 1.2 mg/dL). The ULN values were determined in a representative Thai population at the Chulalongkorn Hospital laboratory 12. All laboratory values were graded according to the U.S. National Institutes of Health Division of AIDS (DAIDS) grading table (version 2.1, July 2017), which grades abnormalities from 1 (mild) to 4 (potentially life‐threatening). Values ≥1.25 times the ULN were considered abnormal in the primary analysis, with values <1.25 times the ULN defined as normal. ALT was the primary parameter of interest. Additional laboratory tests included measurement of plasma soluble immune markers using a combination of standard single‐marker ELISAs and Luminex‐based multiplex panels (Table 2). ELISAs included soluble CD14 (R&D Systems, Minneapolis MN), neopterin (Genway Biotech, San Diego, CA), and high‐sensitivity IFN‐α (PBL Assay Science, Piscataway, NJ). Custom configured Luminex‐based multiplex panels from multiple manufacturers were utilized for the remaining assays (MilliporeSigma, Burlington, MA; Bio‐Rad Laboratories, Hercules, CA; and ThermoFisher Scientific, Waltham, MA).

Participants were considered to manifest acute retroviral syndrome (ARS) if they demonstrated at least three clinical features consistent with this diagnosis 13. Alcohol and drug use data were collected as binary variables measuring any use in the previous four months in a baseline questionnaire at the time of acute HIV diagnosis.

Statistical analyses included descriptive statistics and non‐parametric correlative analyses. The Mann‐Whitney test was used to compare characteristics between the sub‐groups with normal and abnormal baseline ALT. Dunn's pairwise comparison was used to compare continuous outcomes across Fiebig stages and ART regimens. Spearman correlation was used to identify the soluble immune markers significantly correlated with clinical measures, with FDR adjustment (BH procedure) for multiple comparisons applied. A multivariate logistic regression model was used to identify factors associated with elevated ALT at baseline. Random effects models were used to identify factors associated with elevated ALT at 24 and 48 weeks. We used Stata 15.0 (StataCorp LP, College Station, TX, USA) and R 3.6.0 (1.1.453, R Consortium, Boston, MA) for statistical analyses. Figures were created using GraphPad Prism 7.0 (GraphPad Software, La Jolla, CA, USA). *P*‐values were adjusted for multiple comparisons using the Bonferroni method where appropriate.

All participants provided informed consent. The cohort study was approved by the Institutional Review Boards at Chulalongkorn University in Bangkok, Thailand, and the Walter Reed Army Institute of Research, MD, USA.

## 3 Results

Demographics of the participants are described in Table 1. Three‐quarters (77.2%) demonstrated symptoms compatible with ARS. The majority of participants (77.5%) were infected with HIV subtype CRF01_AE. The median CD4^+^ T cell count was 364 cells/μL and median plasma HIV RNA was 5.9 log_10_ copies/mL.

**Table 1:** Baseline characteristics of participants

Characteristics	Overall (n = 426)	Normal ALT (n = 360)	Abnormal ALT (n = 66)	*p*‐value
Age, median years (IQR)	25 (22 to 30)	25 (22 to 30)	25 (22 to 30)	0.871
Risk group, n (%)				
MSM	399 (93.7)	335 (93.1)	64 (97.0)	0.359
Heterosexual male	15 (3.5)	13 (3.6)	2 (3.0)	
Heterosexual female	12 (2.8)	12 (3.3)	–	
Education level, n (%)				
Primary school or lower	11 (2.6)	10 (2.8)	1 (1.5)	0.957
Secondary school	136 (31.9)	116 (32.2)	20 (30.3)	
Diploma	30 (7.0)	26 (7.2)	4 (6.1)	
Bachelor degree or higher	249 (58.5)	208 (57.8)	41 (62.1)	
HIV subtype, n (%)				
CRF01_AE	330 (77.5)	281 (78.1)	49 (74.2)	0.476
B	11 (2.6)	10 (2.8)	1 (1.5)	
01AE/B	61 (14.3)	49 (13.6)	12 (18.2)	
Other	2 (0.5)	1 (0.3)	1 (1.5)	
Nontypeable/Unknown	22 (5.2)	19 (5.3)	3 (4.6)	
Fiebig stage, n (%)				
I	61 (14.3)	59 (16.4)	2 (3.0)	<0.001
II	106 (24.9)	100 (27.8)	6 (9.1)	
III	180 (42.2)	141 (39.2)	39 (59.1)	
IV	51 (12.0)	40 (11.1)	11 (16.7)	
V	28 (6.6)	20 (5.6)	8 (12.1)	
VDRL+, n/N (%)	51/400 (12.8)	43/336 (12.8)	8/64 (12.5)	1.000
Alcohol use, n (%)	98 (23)	81 (23)	17 (26)	0.633
Drug use, n (%)	85 (20.0)	71 (20.0)	14 (21.2)	0.741
ARS Present, n (%)	329 (77.2)	267 (74.2)	62 (93.9)	<0.001
CD4 count (cells/mm^3^), median (IQR)	364 (265 to 495)	368 (266 to 495)	354 (231 to 515)	0.394
<200	43 (10.1)	32 (8.9)	11 (16.7)	0.204
200 to 349	155 (36.4)	135 (37.5)	20 (30.3)	
350 to 500	124 (29.1)	107 (29.7)	17 (25.8)	
>500	104 (24.4)	86 (23.9)	18 (27.3)	
CD4/CD8 ratio, median (IQR)	0.70 (0.43 to 1.04)	0.77 (0.49 to 1.11)	0.42 (0.20 to 0.61)	<0.001
CD4/CD8 ratio> 1	117 (27.5)	111 (30.8)	6 (9.1)	<0.001
HIV‐RNA (log_10_copies/mL), median (IQR)	5.9 (5.3 to 6.7)	5.8 (5.2 to 6.6)	6.4 (5.8 to 6.9)	<0.001
<100,000	83 (19.5)	79 (21.9)	4 (6.1)	<0.001
100,000 to 999,999	148 (34.7)	131 (36.4)	17 (25.8)	
≥1,000,000	195 (45.8)	150 (41.7)	45 (68.2)	
Initial antiretroviral therapy				
EFV/TDF/XTC	288 (67.6)	243 (67.5)	45 (68.2)	0.644
EFV/TDF/XTC + RAL/MVC	78 (18.3)	66 (18.3)	12 (18.2)	
DTG/TDF/XTC	26 (6.1)	22 (6.1)	4 (6.1)	
DTG/TDF/XTC + MVC	25 (5.9)	22 (6.1)	3 (4.6)	
EFV/TDF/FTC + Telmisartan	6 (1.4)	5 (1.4)	1 (1.5)	
RAL/TDF/3TC	1 (0.2)	1 (0.3)	–	
TDF/FTC + MVC/RAL	1 (0.2)	1 (0.3)	–	
EFV/TDF/3TC + MVC	1 (0.2)	–	1 (1.5)	
Degree of ALT elevation				
Grade 1 (1.25 to < 2.5 × ULN)	–	–	43 (65.2)	
Grade 2 (2.5 to < 5.0 × ULN)	–	–	15 (22.7)	
Grade 3 (5.0 to < 10.0 × ULN)	–	–	8 (12.1)	
Grade 4 (≥10.0 × ULN)	–	–	0 (0)	

EFV, efavirenz; IQR, interquartile range; MSM, men who have sex with men; MVC, maraviroc; RAL, raltegravir; TDF, tenofovir disoproxil fumarate; VDRL, venereal disease research laboratory test for syphilis; XTC, lamivudine (3TC) or emtricitabine (FTC).

### 3.1 Elevated ALT levels are common in acute HIV infection and are more common in Fiebig stages III to V

Sixty‐six of the 426 individuals (15.5%) exhibited elevated baseline ALT levels (Table 1). Of those with abnormal baseline ALT, the majority (43/66, 65.2%) had DAIDS Grade 1 elevations (between 1.25 and 2.5 times the ULN). Twenty‐three of the 66 individuals (34.8%) had Grade 2 to 3 elevations in baseline ALT, and no participants had Grade 4 elevations at baseline.

When comparing participants with normal (n = 360) and abnormal (n = 66) baseline ALT levels, there was no difference in age, risk group, education level, HIV subtype, syphilis seropositivity, alcohol use, recreational drug use or baseline CD4^+^ T lymphocyte count (Table 1). CD4/CD8 ratio was lower in individuals with elevated ALT levels.

### 3.2 Correlates of elevated ALT at baseline

**Figure 1:**
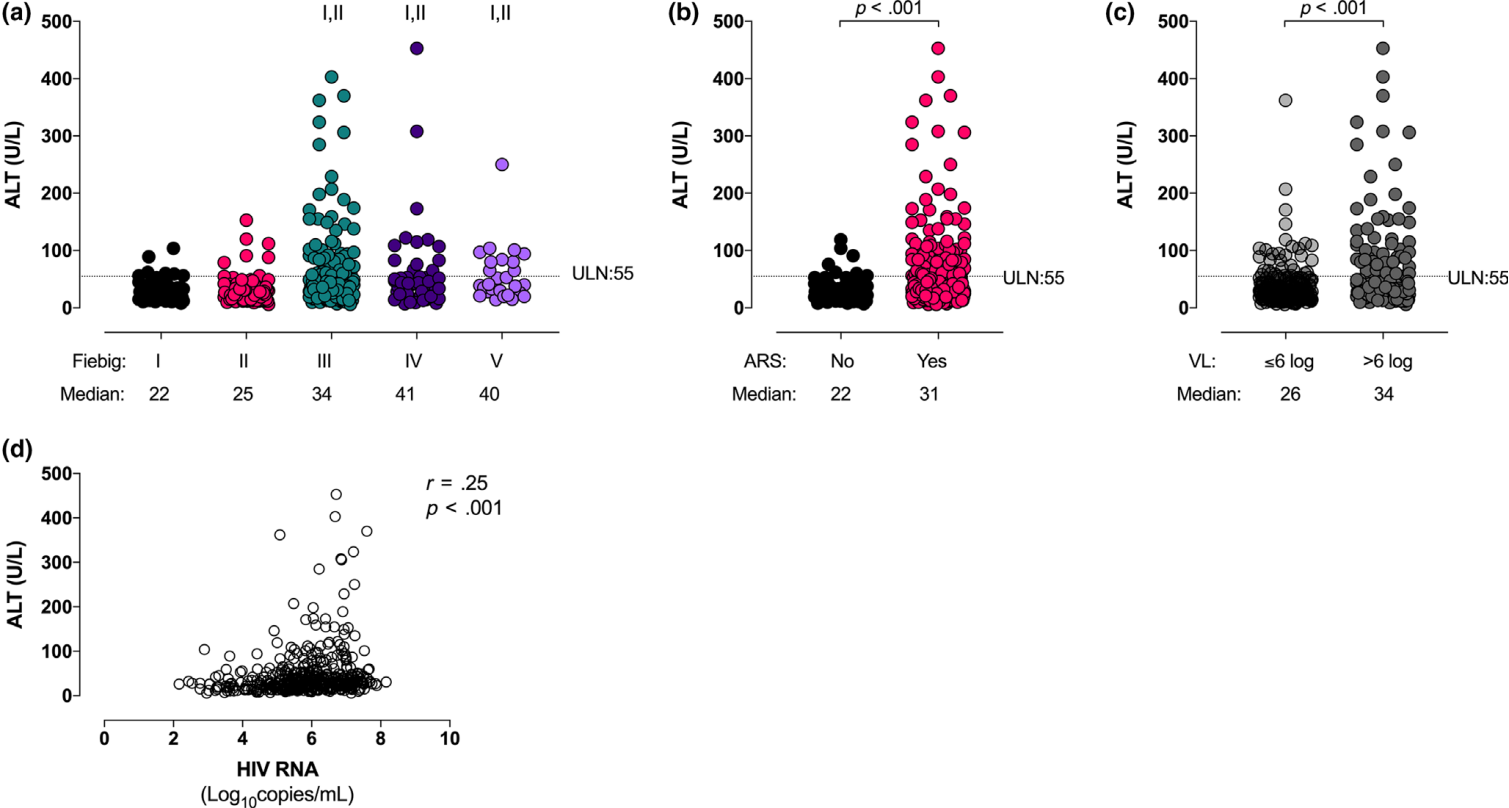
(**a**) ALT levels stratified by Fiebig stage at diagnosis. (**b**) ALT levels stratified by the presence or absence of acute retroviral syndrome at diagnosis. (**c**) ALT levels stratified by HIV plasma RNA level at diagnosis. (**d**) ALT correlates with HIV RNA. I indicates statistically significant compared with Fiebig I, II indicates statistically significant compared with Fiebig II.

Individuals enrolled during Fiebig stages I to II had lower baseline ALT compared with those enrolled during Fiebig stages III to V (Table 1, Figure 1a). Those with elevated ALT were more likely to be experiencing ARS than those with normal baseline ALT (93.9% vs. 74.2%, *p* < 0.001; Table 1), and most Grade 2 to 4 elevations occurred in those experiencing ARS (Figure 1b).

Those with higher baseline plasma HIV RNA had higher baseline ALT (Figure 1c), and baseline ALT levels correlated with plasma HIV RNA values (r = 0.25, *p* < 0.001; Figure 1d). In a multivariate model incorporating age, Fiebig stage, alcohol and drug use, ARS, CD4^+^ T lymphocyte count, and plasma HIV RNA, only Fiebig stages III to V and baseline HIV RNA >6 log_10_ copies/mL were independently associated with elevated baseline ALT levels (Table S1; OR = 3.96, *p* = 0.001 and OR = 2.12, *p* = 0.012 respectively). In a subsequent model limited to Grade 2 or greater ALT elevations (n = 23), both Fiebig stages III to V and baseline HIV RNA >6 log_10_ copies/mL remained independently associated with elevated baseline ALT levels (OR 11.2, *p* = 0.02 and OR = 4.4, *p* = 0.0008 respectively).

In a sub‐analysis of participants with plasma soluble marker information available, baseline ALT levels correlated with soluble tumour necrosis factor (TNF) receptor II (r = 0.35, *p* < 0.001), soluble CD30 (r = 0.38, *p* = 0.004), TNF‐alpha (r = 0.23, *p* = 0.01), soluble CD163 (r = 0.22, *p* = 0.018), T‐cell immunoglobulin and mucin domain (Tim)‐3 (r = 0.26, *p* = 0.033) and interferon gamma‐induced protein (IP)‐10 (r = 0.19, *p* = 0.048; Table 2).

**Table 2:** Baseline correlations between LFTs and plasma soluble markers

Clinical measure	Marker	n	Spearman r	*p*‐value (adj)
Baseline	TNF‐RII	174	0.35	<0.001
ALT	CD30	82	0.38	0.004
	TNF‐α	212	0.23	0.009
	CD163	189	0.22	0.018
	TIM‐3	124	0.26	0.033
	IP10	212	0.19	0.048
Baseline	TNF‐RII	173	0.34	<0.001
GGT	IP10	211	0.23	0.010
	CD30	82	0.36	0.011
	RANTES	182	−0.22	0.026
	TIM‐3	123	0.26	0.036
	CD27	123	0.25	0.038
Baseline	Neopterin	206	−0.36	<0.001
Bilirubin	IP10	212	−0.33	<0.001
	MCP‐1	212	−0.30	<0.001
	CD14	211	−0.28	0.001
	IFN‐α	206	−0.28	0.001
	VEGF‐D	33	−0.48	0.035

ALT, alanine aminotransferase; GGT, gamma‐glutamyl transferase; IFN, interferon; IP, interferon gamma‐induced protein; MCP, monocyte chemoattractant protein; RANTES, regulated on activation, normal T cell expressed and secreted; TIM, T‐cell immunoglobulin and mucin domain; TNF, tumor necrosis factor.

### 3.3 Longitudinal trends in ALT

**Figure 2:**
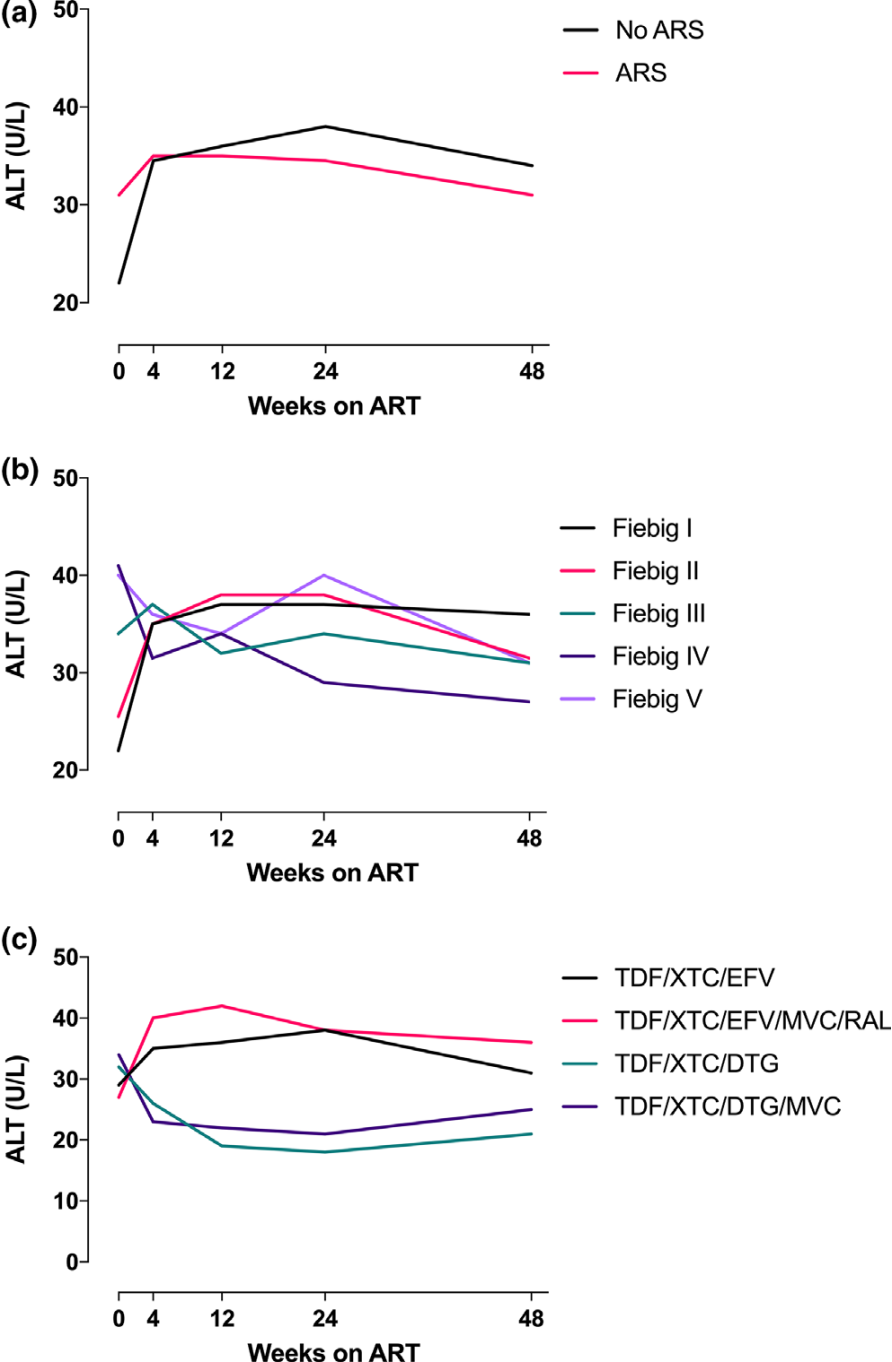
(**a**) Longitudinal ALT levels to 48 weeks according to the presence or absence of acute retroviral syndrome at diagnosis. (**b**) Longitudinal ALT levels to 48 weeks by Fiebig stage at diagnosis. (**c**) Longitudinal ALT levels according to ART regimen initiated at the time of diagnosis.

While those with ARS had higher median ALT at baseline than those without this syndrome (31 vs. 22 U/L, *p* < 0.001), this difference resolved by four weeks after ART initiation (Figure 2a). After week 4, individuals who had not experienced ARS tended to have slightly higher ALT levels, although the difference was not statistically significant (Figure 2a).

Between baseline and week 24, the median ALT level increased in individuals diagnosed during Fiebig stages I to II and was stable or decreased in individuals diagnosed during Fiebig stages III to V (Figure 2b). At week 24, elevations in ALT were predicted by Fiebig stages I to II at enrolment (OR 24.8, *p* < 0.001), abnormal baseline ALT (OR 55.6, *p* < 0.001), and baseline plasma HIV RNA less than 6 log_10_ copies/mL (OR 21.9, *p* < 0.001) in a multivariate model (Table S2).

Trends in ALT levels differed according to ART regimen initiated at baseline, with those initiating efavirenz‐based regimens having an initial increase in ALT and those initiating non‐efavirenz‐based regimens experiencing an initial decline in ALT (Figure 2c). Individuals who had been initiated on non‐efavirenz‐based ART were less likely to have elevated ALT at 48 weeks compared with individuals on efavirenz‐based regimens (Table S3; *p* = 0.003). Other factors associated with week 48 ALT elevation included older age, abnormal baseline ALT and baseline CD4 count >350 cells/μL (Table S3).

### 3.4 Baseline ALT as a predictor of clinical outcome

We investigated clinical outcomes (virologic suppression, CD4^+^ T lymphocyte count, change in CD4^+^ T cell count and CD4/CD8 T lymphocyte ratio) according to the presence of abnormal baseline ALT. There was a trend towards association between abnormal baseline ALT levels and lower rates of virologic suppression (defined as plasma HIV RNA <50 copies/mL) at week 4 (19.4% vs. 9.2%, *p* = 0.05), week 12 (64.9% vs. 46.2%, *p* = 0.08) and week 24 (95.5% vs. 87.9%, *p* = 0.06), even when adjusted for the baseline plasma HIV RNA value; this trend was no longer present at week 48 (98.3% vs. 97%, *p* = 0.37). There was no association between baseline ALT abnormality and CD4^+^ T cell count, magnitude of CD4^+^ T cell change from baseline, or CD4/CD8 ratio at week 4, 12, 24 or 48 follow‐up time points.

### 3.5 Three year trends in LFTs

**Figure 3:**
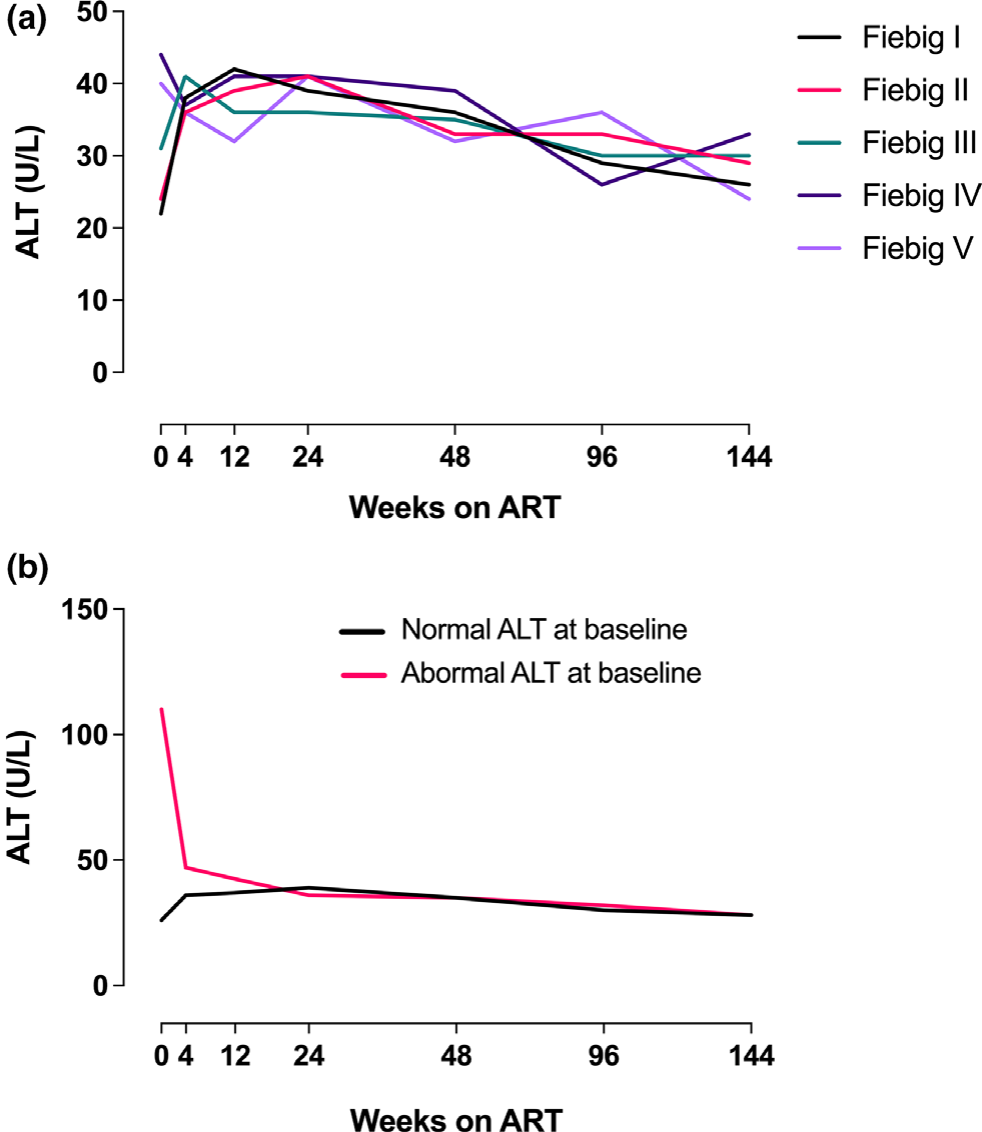
Trends to 144 weeks based upon (**a**) Fiebig stage at diagnosis and (**b**) presence of ALT elevations at baseline.

We examined LFT trends to week 96 and week 144 in a subset of individuals (n = 278 and n = 282 respectively) with longitudinal data available at these time points. There were no clear trends after the first year on ART based upon initial Fiebig stage (Figure 3a). When the sample was stratified by the presence or absence of abnormal baseline ALT, the ALT differences between groups resolved in the first 12 weeks of therapy and then remained normal through 144 weeks (Figure 3b).

### 3.6 GGT elevations, correlates and longitudinal trends

**Figure 4:**
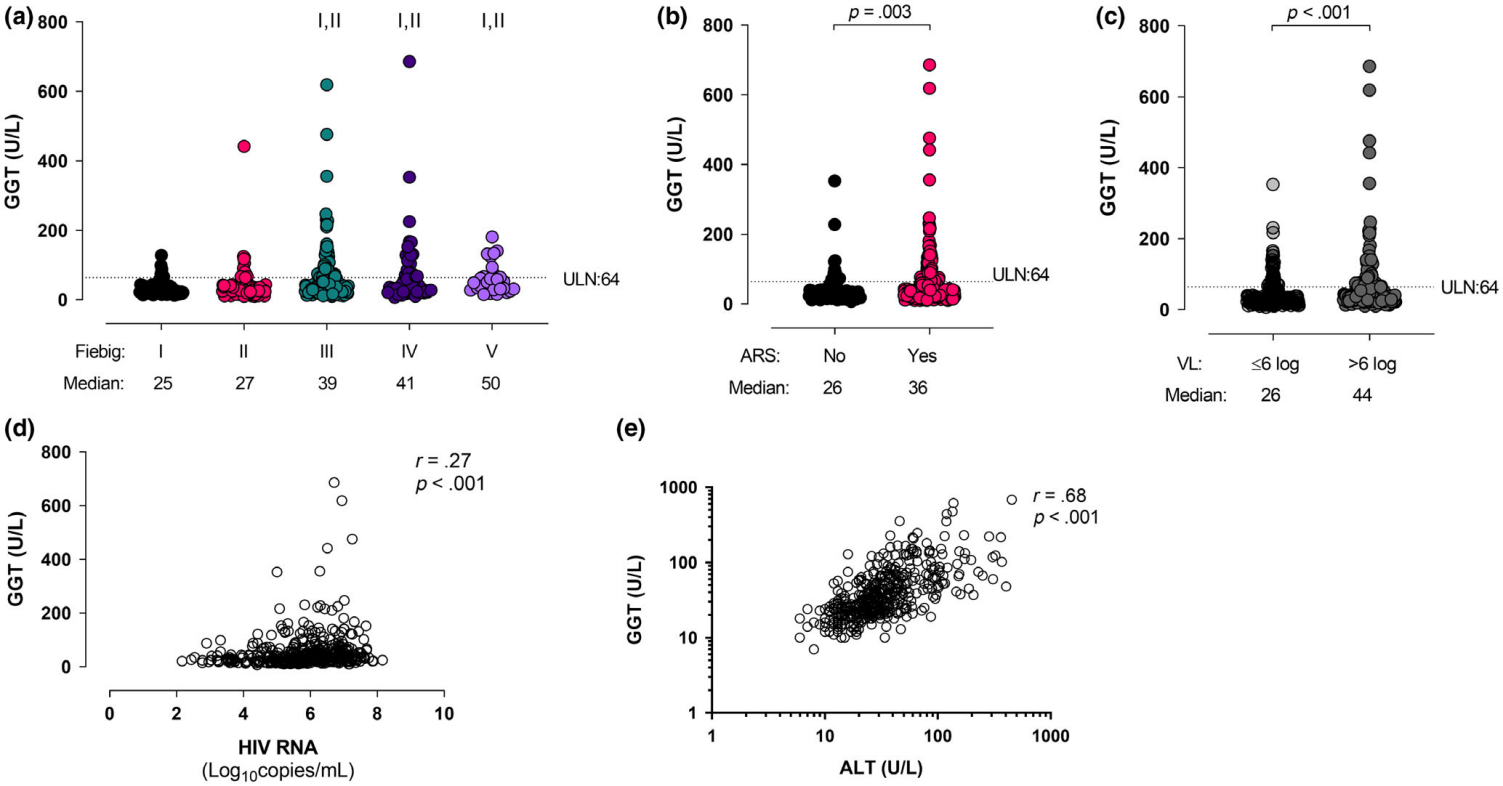
(**a**) GGT levels stratified by Fiebig stage at diagnosis. (**b**) GGT levels stratified by the presence or absence of acute retroviral syndrome at diagnosis. (**c**) GGT levels stratified by HIV plasma RNA level at diagnosis. (**d**) GGT correlates with HIV RNA at baseline. (**e**) GGT correlates with ALT at baseline. I indicates statistically significant compared with Fiebig I, II indicates statistically significant compared with Fiebig II.

Sixty‐six of the 425 individuals (15.5%) had elevated GGT at baseline. As with ALT, the greatest proportion and highest magnitude of GGT elevation was seen in Fiebig stages III to V (Figure 4a) and in those with ARS (Figure 4b). There was a weak correlation between baseline GGT and plasma HIV RNA (r = 0.27, *p* < 0.001; Figure 4d) and a strong correlation between baseline ALT and GGT (r = 0.68, *p* < 0.001; Figure 4e).

Baseline GGT correlated with soluble TNF receptor II (r = 0.34, *p* < 0.001), IP‐10 (r = 0.23, *p* = 0.01), soluble CD30 (r = 0.36, *p* = 0.01), Tim‐3 (r = 0.26, *p* = 0.036) and CD27 (r = 0.25, *p* = 0.038). An inverse correlation with the protein regulated on activation, normal T cell expressed and secreted (RANTES; r = −0.22, *p* = 0.03) was also identified (Table 2).

The presence of GGT abnormalities at baseline was predictive of GGT abnormalities at weeks 4, 12, 24 and 48 follow‐up time points on ART, although the magnitude of this association decreased over time (data not shown).

### 3.7 Total bilirubin elevation is uncommon and inversely correlates with HIV RNA

**Figure 5:**
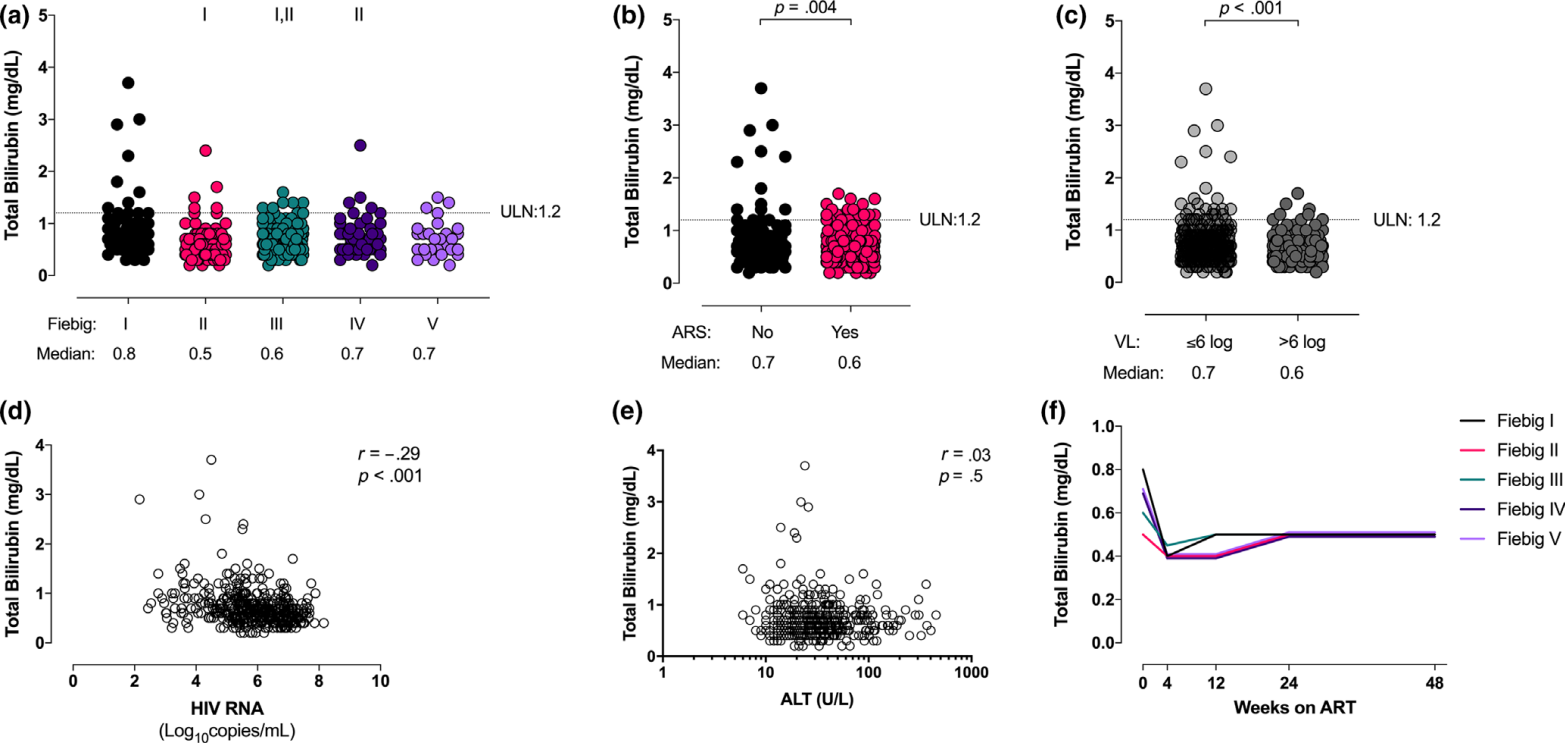
(**a**) Total bilirubin levels stratified by Fiebig stage at diagnosis. (**b**) Total bilirubin levels stratified by the presence or absence of acute retroviral syndrome at diagnosis. (**c**) Total bilirubin levels stratified by HIV plasma RNA level at diagnosis. (**d**) Total bilirubin inversely correlates with HIV RNA. (**e**) Total bilirubin does not correlate with ALT at baseline. (**f**) Longitudinal total bilirubin levels to 48 weeks by Fiebig stage at diagnosis. I indicates statistically significant compared with Fiebig I, II indicates statistically significant compared with Fiebig II.

Nineteen of the 426 individuals (4.5%) had elevated total bilirubin at baseline. Interestingly, most bilirubin elevations occurred during Fiebig stages I to II (Figure 5a), in those not experiencing ARS (Figure 5b), and in those with baseline plasma HIV RNA <6 log_10_ copies/mL (Figure 5c). Total bilirubin showed an inverse relationship with plasma HIV RNA (r = −0.29, *p* < 0.01; Figure 5d), but did not correlate with ALT (Figure 5e) or GGT (not shown).

Baseline total bilirubin demonstrated inverse correlations with neopterin (r=−0.36, *p* < 0.001), IP‐10 (r=−0.33, *p* < 0.001), monocyte chemoattractant protein (MCP)‐1 (r=−0.30, *p* < 0.001), soluble CD14 (r=−0.28, *p* = 0.001), interferon‐alpha (r=−0.28, *p* = 0.001) and vascular endothelial growth factor (VEGF)‐D (r=−0.48, *p* = 0.035; Table 2).

Over time, bilirubin tended to decrease and then plateau regardless of the Fiebig stage at diagnosis (Figure 5f).

## 4 Discussion

Elevation in ALT levels was a common feature of AHI in this large study cohort. There were significant differences in LFT trends within the different Fiebig stages and between different ART regimens. Elevations in ALT and decreases in total bilirubin correlated with plasma HIV RNA and plasma markers of inflammation, including markers of monocyte/macrophage activation. Importantly, most ALT elevations detected at baseline resolved over time with ART.

While LFT abnormalities were common, we detected elevated ALT in a lower proportion of individuals than has been described previously. Analysis of a small Chinese cohort (n = 23) found that 65% of individuals with primary HIV infection had elevated ALT levels 6. A cross‐sectional study of a prospectively enrolled Swiss cohort found that 61% of individuals with ARS had elevated ALT levels 7. Methodologic differences, including limited viral hepatitis testing, lack of or different distribution of Fiebig staging, and different case definitions for ALT elevation in the prior reports make it difficult to directly compare these proportions. It is also likely that we were able to identify a larger proportion of individuals in early Fiebig stages who had not yet developed abnormalities before initiation of ART. This analysis therefore provides additional perspective on the frequency of ALT elevations in very early HIV infection, and on the potential benefits of early ART initiation during AHI.

There were several notable trends with regard to ALT elevations in this cohort. At baseline, elevated ALT levels concentrated in individuals diagnosed in later Fiebig stages, when the HIV viral load peaks and the immune response is most active. At later time points (week 24 and week 48), ALT elevations were predicted by higher baseline ALT, earlier Fiebig stages at enrolment, lower baseline HIV RNA and higher baseline CD4^+^ T lymphocyte count, and ART regimen. Longitudinal trends showed that individuals diagnosed in Fiebig stages III to V tended to have stable or downtrending ALT levels following ART initiation. Individuals diagnosed in earlier Fiebig stages tended to have a subsequent increase in ALT levels despite early ART initiation. In general, these elevations resolved. Further understanding of these trends is important in the light of a recent study that demonstrated that most biopsies performed for transaminase elevations in HIV‐monoinfected individuals showed nonspecific pathology 14.

Taken together, our findings suggest a multifactorial model for hepatic injury involving a combination of HIV‐associated and ART‐associated processes. HIV‐associated hepatocellular injury due to immune‐associated processes may be the dominant process driving ALT perturbations in later Fiebig stages, causing baseline elevation with subsequent normalization of ALT levels. Earlier diagnosis and ART initiation in Fiebig stages I to II may prevent the HIV‐associated hepatocellular injury that arises in later stages of AHI, but may also preserve vulnerable hepatocytes (i.e. those that are most susceptible) for injury due to drug toxicity, a slower process that becomes apparent over the first six to twelve months of ART 11. While most abnormalities resolve over time, in a small subset, perturbations may be slow to recover or not completely reversible.

A multifactorial model involving differential risk to HIV‐associated and ART‐associated hepatic effects is supported by several observations. First, a significant proportion of those treated in earlier Fiebig stages do not develop the robust immune response that characterizes the later stages 15. This makes it less likely that a volunteer diagnosed in Fiebig I to II would “progress” to Fiebig III to V and develop LFT abnormalities due to immune‐associated processes (e.g. processes related to inflammation or cell activation). Second, the association of efavirenz‐based ART regimens with ALT elevations at 48 weeks indicates that ART‐associated liver injury takes time to develop, and that individuals treated during very early infection may experience this because of the mitigation of early immune‐associated injury and preservation of vulnerable hepatocytes. This suggests that both HIV‐associated viral and immunologic effects as well as ART‐associated effects can drive ALT perturbations at different stages in acute HIV infection and through the first 48 weeks of ART.

This analysis provides some clues as to potential mechanisms driving HIV‐associated hepatic injury. A previous study found a correlation of similar magnitude between plasma HIV RNA and elevated ALT in a smaller cohort (n = 59), and hypothesized that this could be related to the apoptotic properties of HIV viral proteins 16. Other studies have hypothesized that HIV results in hepatocellular damage through cellular activation, although data on its potential to infect hepatocytes and Kuppfer cells are limited 4, 17, 18. Recent work using a simian immunodeficiency virus model in macaques suggested that macrophage and T cell activation were primary drivers of hepatic inflammation 19. In our analysis, ALT levels correlated with soluble CD163, a marker specific to activated macrophages that has been associated with Kupffer cell activation 20, 21. However, it is also possible that hepatocellular injury could be a feature of the systemic immune activation seen in response to both the virus itself and following T cell depletion in the gut, which is known to result in mucosal leakage and microbial translocation 4, 18, 22. ALT levels also correlated with soluble TNF‐alpha, a non‐specific marker of immune activation, and Tim‐3, a marker which is expressed on activated CD4^+^ and CD8^+^ T lymphocytes, regulatory T cells, and innate immune cells. In chronic HIV, the Tim‐3 receptor may be associated with T cell dysfunction and exhaustion 23.

In this cohort, baseline total bilirubin levels inversely correlated with markers of general inflammation (interferon alpha, VEGF), macrophage activation (neopterin, soluble CD14) and chemokines targeting macrophages and lymphocytes (IP‐10, MCP‐1). In addition, regardless of Fiebig stage, total bilirubin decreased between baseline and week 4 before plateauing. Bilirubin is known to have anti‐inflammatory effects and may reduce oxidative stress, and recent data have shown that hyperbilirubinemia is associated with decreased cardiovascular disease risk in PLHIV 24. The correlations and longitudinal trends described here might reflect these anti‐inflammatory properties, and possibly suggest that immune re‐equilibration following initial HIV infection may occur even with ART initiation. Further work will be necessary to understand the clinical significance of these changes, as bilirubin has been proposed as a potential biomarker of inflammation‐mediated conditions in PLHIV 24. This could include exploring longitudinal outcomes in individuals with different bilirubin nadirs during acute infection to determine whether these levels have an impact on HIV disease progression, general markers of inflammation, or downstream consequences of systemic inflammation (liver, kidney or cardiovascular disease).

This study had several notable limitations. While the sample size was large, the cohort examined in this study is relatively homogeneous and comprised of young Asian MSM without significant comorbidities. These participants therefore might not be representative of the general population and may also demonstrate greater physiological reserve or a more robust inflammatory response than older individuals and those with intercurrent illnesses. The clinical significance of small differences in LFTs is also unclear. For example, although differences in median baseline ALT levels between those with and without ARS were statistically different, the median in those with ARS was still within the normal range. The clinical significance of these findings is supported by the fact that most abnormal values (i.e. Grade 2 or greater abnormalities) tended to be associated with certain characteristics (Fiebig stage III, ARS, high plasma HIV RNA). Furthermore, even small differences between groups may provide clues regarding HIV pathogenesis and the mechanisms by which the virus affects hepatocytes.

## 5 Conclusions

We have shown that abnormal serum LFTs are common during acute HIV infection in our population and are associated with the stage of infection and markers of inflammation. These data provide insight into potential mechanisms of liver injury during the first year of infection and demonstrate that most individuals do well from this perspective following ART initiation. They also support testing for HIV infection in the setting of a viral illness with associated LFT perturbations. Further research is necessary to better determine the mechanisms that underlie LFT elevations in AHI (e.g. whether hepatocytes or Kuppfer cells are directly involved, and whether the mechanism of injury differs over the course of early infection), their relationship to the systemic inflammatory process, and their prognostic implications.

## Competing interests

JA has received honoraria for participating in advisory meetings for ViiV Healthcare, Gilead, Merck, Roche and AbbVie. SS directs a study through the AIDS Clinical Trials Group for which study medications are provided by Viiv Healthcare.

## Authors' contributions

EK, JA, SS, NP, PP, MLR, DJC and MJP designed the study. EK, JA and NP led the study, with management support from DJC, JI, NC and PP. MJP, SP and NJ designed and performed the statistical analyses. SU, RT, BMS and SJK performed and interpreted laboratory assays. MJP and EK drafted the manuscript, which was reviewed and approved by all other authors.

## Supporting information


**Table S1.** Factors associated with ALT>1.25 ULN at baseline
**Table S2.** Factors associated with ALT>1.25 ULN at 24 weeks
**Table S3.** Factors associated with ALT>1.25 ULN at 48 weeks

## References

[1] Antiretroviral Therapy Cohort Collaboration . Causes of death in HIV‐1‐infected patients treated with antiretroviral therapy, 1996–2006: collaborative analysis of 13 HIV cohort studies. Clin Infect Dis. 2010;50(10):1387–96. 20380565 10.1086/652283 PMC3157754

[2] Rivero A , Mira JA , Pineda JA . Liver toxicity induced by non‐nucleoside reverse transcriptase inhibitors. J Antimicrob Chemother. 2007;59(3):342–6. 17255142 10.1093/jac/dkl524

[3] Price JC , Thio CL . Liver disease in the HIV‐infected individual. Clin Gastroenterol Hepatol. 2010;8(12):1002–12. 20851211 10.1016/j.cgh.2010.08.024 PMC2997131

[4] Sherman KE , Peters MG , Thomas DL . HIV and the liver. Top Antivir Med. 2019;27(3):101–10. 31634861 PMC6892621

[5] Seth A , Sherman KE . Fatty liver disease in persons with HIV infection. Top Antivir Med. 2019;27(2):75–82. 31136997 PMC6550355

[6] Chen YJ , Tsai HC , Cheng MF , Lee SS , Chen YS . Primary human immunodeficiency virus infection presenting as elevated aminotransferases. J Microbiol Immunol Infect. 2010;43(3):175–9. 21291843 10.1016/S1684-1182(10)60028-X

[7] Braun DL , Kouyos R , Oberle C , Grube C , Joos B , Fellay J , et al. A novel Acute Retroviral Syndrome Severity Score predicts the key surrogate markers for HIV‐1 disease progression. PLoS ONE. 2014;9:e114111. 25490090 10.1371/journal.pone.0114111 PMC4260784

[8] Ananworanich J , Schuetz A , Vandergeeten C , Sereti I , de Souza M , Rerknimitr R , et al. Impact of multi‐targeted antiretroviral treatment on gut T cell depletion and HIV reservoir seeding during acute HIV infection. PLoS ONE. 2012;7:e33948. 22479485 10.1371/journal.pone.0033948 PMC3316511

[9] Ananworanich J , Fletcher JL , Pinyakorn S , van Griensven F , Vandergeeten C , Schuetz A , et al. A novel acute HIV infection staging system based on 4th generation immunoassay. Retrovirology. 2013;10:56. 23718762 10.1186/1742-4690-10-56 PMC3669623

[10] Fiebig EW , Wright DJ , Rawal BD , Garrett PE , Schumacher RT , Peddada L , et al. Dynamics of HIV viremia and antibody seroconversion in plasma donors: implications for diagnosis and staging of primary HIV infection. AIDS. 2003;17(13):1871–9. 12960819 10.1097/00002030-200309050-00005

[11] Deshwal R , Arora S . Serum alanine aminotransferase elevations in HIV positive patients on antiretroviral therapy in India. J Assoc Physicians India. 2019;67(3):67–70. 31304710

[12] Chamnanpai S , Charuruks N , Watanaboonyoungcharoen P , Kalayanachati A . Reference intervals of clinical chemistry parameters in adults at King Culalongkorn Memorial Hospital. Chula Med J. 2004;48(8):521–9.

[13] Crowell TA , Colby DJ , Pinyakorn S , Fletcher JLK , Kroon E , Schuetz A , et al. Acute retroviral syndrome is associated with high viral burden, CD4 depletion, and immune activation in systemic and tissue compartments. Clin Infect Dis. 2018;66(10):1540–9. 29228130 10.1093/cid/cix1063 PMC5930255

[14] Iogna Prat L , Roccarina D , Lever R , Lombardi R , Rodger A , Hall A , et al. Etiology and severity of liver disease in HIV‐positive patients with suspected NAFLD: lessons from a Cohort with available liver biopsies. J Acquir Immune Defic Syndr. 2019;80(4):474–80. 30807482 10.1097/QAI.0000000000001942

[15] de Souza MS , Pinyakorn S , Akapirat S , Pattanachaiwit S , Fletcher JL , Chomchey N , et al. Initiation of antiretroviral therapy during acute HIV‐1 infection leads to a high rate of nonreactive HIV serology. Clin Infect Dis. 2016;63(4):555–61. 27317797 10.1093/cid/ciw365

[16] Mata‐Marin JA , Gaytan‐Martinez J , Grados‐Chavarria BH , Fuentes‐Allen JL , Arroyo‐Anduiza CI , Alfaro‐Mejia A . Correlation between HIV viral load and aminotransferases as liver damage markers in HIV infected naive patients: a concordance cross‐sectional study. Virology. 2009;6:181. 10.1186/1743-422X-6-181 PMC2777159 19878552

[17] Cao YZ , Dieterich D , Thomas PA , Huang YX , Mirabile M , Ho DD . Identification and quantitation of HIV‐1 in the liver of patients with AIDS. AIDS. 1992;6(1):65–70. 1543567 10.1097/00002030-199201000-00008

[18] Ganesan M , Poluektova LY , Kharbanda KK , Osna NA . Liver as a target of human immunodeficiency virus infection. World J Gastroenterol. 2018;24(42):4728–37. 30479460 10.3748/wjg.v24.i42.4728 PMC6235802

[19] Fisher BS , Green RR , Brown RR , Wood MP , Hensley‐McBain T , Fisher C , et al. Liver macrophage‐associated inflammation correlates with SIV burden and is substantially reduced following cART. PLoS Pathog. 2018;14:e1006871. 29466439 10.1371/journal.ppat.1006871 PMC5837102

[20] Gronbaek H , Sandahl TD , Mortensen C , Vilstrup H , Moller HJ , Moller S . Soluble CD163, a marker of Kupffer cell activation, is related to portal hypertension in patients with liver cirrhosis. Aliment Pharmacol Ther. 2012;36(2):173–80. 22591184 10.1111/j.1365-2036.2012.05134.x

[21] Hiraoka A , Horiike N , Akbar SM , Michitaka K , Matsuyama T , Onji M . Expression of CD163 in the liver of patients with viral hepatitis. Pathol Res Pract. 2005;201(5):379–84. 16047947 10.1016/j.prp.2004.10.006

[22] Brenchley JM , Price DA , Schacker TW , Asher TE , Silvestri G , Rao S , et al. Microbial translocation is a cause of systemic immune activation in chronic HIV infection. Nat Med. 2006;12(12):1365–71. 17115046 10.1038/nm1511

[23] Finney CA , Ayi K , Wasmuth JD , Sheth PM , Kaul R , Loutfy M , et al. HIV infection deregulates Tim‐3 expression on innate cells: combination antiretroviral therapy results in partial restoration. J Acquir Immune Defic Syndr. 2013;63(2):161–7. 23314411 10.1097/QAI.0b013e318285cf13

[24] Marconi VC , Duncan MS , So‐Armah K , Re VL , Lim JK , Butt AA , et al. Bilirubin Is inversely associated with cardiovascular disease among HIV‐positive and HIV‐negative individuals in VACS (Veterans Aging Cohort Study). J Am Heart Assoc. 2018;7:e007792. 29720501 10.1161/JAHA.117.007792 PMC6015337

