# Supplementary material for: Liver function test abnormalities in a longitudinal cohort of Thai individuals treated since acute HIV infection
Source: J Int AIDS Soc. 2020 Jan 17;23(1):e25444. doi: 10.1002/jia2.25444 (PMC6968973; doi:10.1002/jia2.25444)
Supplement: Supplementary file 1 — Table S1. Factors associated with ALT>1.25 ULN at baseline Table S2. Factors associated with ALT>1.25 ULN at 24 weeks Table S3. Factors associated with ALT>1.25 ULN at 48 weeks [file JIA2-23-e25444-s001.docx]

| **Factor** | **Univariate** | | **Multivariate** | |
| --- | --- | --- | --- | --- |
|  | **Odds ratio**  **(95%CI)** | **p-value** | **Adjusted Odds ratio (95%CI)** | **p-value** |
| Age | 1.01 (0.97 – 1.05) | 0.61 |  |  |
| Fiebig stage |  |  |  |  |
| I-II | Ref. |  | Ref. |  |
| III-V | 5.74 (2.66 – 12.36) | <0.001 | 3.96 (1.77 – 8.84) | 0.001 |
| Alcohol use |  |  |  |  |
| No | Ref. |  |  |  |
| Yes | 1.20 (0.65 – 2.19) | 0.56 |  |  |
| Drug use |  |  |  |  |
| No | Ref. |  |  |  |
| Yes | 1.10 (0.58 – 2.09) | 0.78 |  |  |
| Having ARS |  |  |  |  |
| No | Ref. |  | Ref. |  |
| Yes | 5.40 (1.91 – 15.25) | 0.001 | 2.28 (0.75 – 6.88) | 0.145 |
| CD4 count (cells/mm^3^) |  |  |  |  |
| <350 | 1.02 (0.60 – 1.73) | 0.93 |  |  |
| ≥350 | Ref. |  |  |  |
| HIV RNA (copies/mL) |  |  |  |  |
| <1,000,000 | Ref. |  | Ref. |  |
| ≥1,000,000 | 3.00 (1.72 – 5.25) | <0.001 | 2.12 (1.18 – 3.79) | 0.012 |
| CD4/CD8 ratio |  |  |  |  |
| >1 | Ref. |  | Ref. |  |
| ≤ 1 | 4.46 (1.87 – 10.62) | <0.001 | 2.19 (0.87 – 5.51) | 0.097 |

**Table S1.** **Factors associated with ALT >1.25 ULN at baseline.**

| **Factors** | **Univariate** | | **Multivariate** | |
| --- | --- | --- | --- | --- |
|  | **Coefficient**  **(95%CI)** | **p-value** | **Adjusted Coefficient (95%CI)** | **p-value** |
| Age | 0.2 (-0.7 to 1.1) | 0.66 |  |  |
| Fiebig stage |  |  |  |  |
| I-II | 32.5 (20.6 – 44.4) | <0.001 | 24.8 (13.5 to 36.1) | <0.001 |
| III-V | Ref. |  | Ref. |  |
| Alcohol use |  |  |  |  |
| No | Ref. |  |  |  |
| Yes | 0.03 (-14.2 to 14.2) | 1.00 |  |  |
| Drug use |  |  |  |  |
| No | Ref. |  |  |  |
| Yes | -9.3 (-24.3 to 5.6) | 0.22 |  |  |
| Baseline ARS |  |  |  |  |
| No | 28.0 (14.0 to 42.1) | <0.001 |  |  |
| Yes | Ref. |  |  |  |
| ALT at week 0 |  |  |  |  |
| Normal | Ref. |  | Ref. |  |
| Abnormal | 58.6 (43.7 – 73.5) | <0.001 | 55.6 (41.3 to 69.8) | <0.001 |
| Baseline CD4 count (cells/mm^3^) |  |  |  |  |
| <350 | 7.1 (-24.3 to 38.5) | 0.66 |  |  |
| ≥350 | Ref. |  |  |  |
| Baseline HIV RNA (copies/mL) |  |  |  |  |
| <1,000,000 | 30.0 (18.3 – 41.7) | <0.001 | 21.9 (10.8 to 33.0) | <0.001 |
| ≥1,000,000 | Ref. |  | Ref. |  |
| First regimen |  |  |  |  |
| Non-EFV | Ref. |  |  |  |
| EFV | 16.58 (-0.67 to 33.82) | 0.059 |  |  |

**Table S2. Factors associated with ALT >1.25 ULN at 24 weeks.**

| **Factors** | **Univariate** | | **Multivariate** | |
| --- | --- | --- | --- | --- |
|  | **Coefficient**  **(95%CI)** | **p-value** | **Adjusted Coefficient (95%CI)** | **p-value** |
| Age | 0.4 (0.1 to 0.8) | 0.01 | 0.2 (0.03 – 0.5) | 0.03 |
| Fiebig stage |  |  |  |  |
| I-II | Ref. |  |  |  |
| III-V | -2.32 (-7.17 to 2.52) | 0.35 |  |  |
| Alcohol use |  |  |  |  |
| No | Ref. |  |  |  |
| Yes | 2.5 (-3.1 to 8.1) | 0.38 |  |  |
| Drug use |  |  |  |  |
| No | Ref. |  |  |  |
| Yes | -0.6 (-6.5 to 5.3) | 0.83 |  |  |
| Baseline ARS |  |  |  |  |
| No | Ref. |  |  |  |
| Yes | 1.2 (-4.4 to 6.9) |  |  |  |
| ALT at week 0 |  |  |  |  |
| Normal | Ref. |  | Ref. |  |
| Abnormal | 83.3 (79.4 to 87.2) | <0.001 | 82.8 (79.0 – 86.7) | <0.001 |
| Baseline CD4 count (cells/mm^3^) |  |  |  |  |
| <350 | Ref. |  | Ref. |  |
| ≥350 | 6.6 (1.3 – 11.9) | 0.02 | 4.4 (0.5 – 8.4) | 0.03 |
| Baseline HIV RNA (copies/mL) |  |  |  |  |
| <1,000,000 | Ref. |  |  |  |
| ≥1,000,000 | 0.5 (-4.3 to 5.2) | 0.84 |  |  |
| First regimen |  |  |  |  |
| Non-EFV | Ref. |  | Ref. |  |
| EFV | 11.5 (4.8 – 18.2) | 0.001 | 6.6 (2.3 – 11.0) | 0.003 |

**Table S3. Factors associated with ALT >1.25 ULN at 48 weeks.**
